# Conditioning open-label placebo: a pilot pharmacobehavioral approach for opioid dose reduction and pain control

**DOI:** 10.1097/PR9.0000000000000828

**Published:** 2020-07-20

**Authors:** Leon Morales-Quezada, Ines Mesia-Toledo, Anayali Estudillo-Guerra, Kevin C. O'Connor, Jeffrey C. Schneider, Douglas J. Sohn, David M. Crandell, Ted Kaptchuk, Ross Zafonte

**Affiliations:** a Department of Physical Medicine and Rehabilitation, Spaulding Rehabilitation Hospital, Harvard Medical School, Boston, MA, USA; b Program in Placebo Studies and Therapeutic Encounter, Beth Israel Deaconess Medical Center, Harvard Medical School, Boston, MA, USA

**Keywords:** Opioids, Open-label placebo, Conditioning, Pain

## Abstract

Opioid consumption for those in comprehensive inpatient rehabilitation units is high because of the complexity of their injuries. Notably, pain in rehabilitation leads to worsened clinical outcomes because of maladaptive behaviors and poor engagement during therapies. It is critical to developing evidence-based pharmacobehavioral interventions. Based on principles of classical conditioning, conditioning open-label placebo (COLP) is a promising approach for reducing opioid use in comprehensive inpatient rehabilitation, and this technique takes advantage of the possibility of association learning and opioid pharmacology to promote evoked placebo-driven analgesia.

**Objectives:**

In this brief report, we evaluate the feasibility of COLP as a pharmacobehavioral intervention to decrease total opioid consumption in patients with pain hospitalized at Spaulding Rehabilitation Hospital.

**Methods:**

Inpatients with spinal cord injury and polytrauma (n = 20) with moderate to severe pain were randomized to receive COLP (n = 10) or treatment-as-usual for 6 consecutive days. Opioid utilization was measured by morphine equivalents using the morphine equivalent dose conversion; pain severity was assessed using the numerical visual analog scale.

**Results:**

Conditioning open-label placebo significantly reduced total opioid consumption by the end of the intervention period (*P* ≤ 0.001). Pain reduction was also significant for the COLP group (*P* = 0.005), whereas the treatment-as-usual group demonstrated a trend towards pain reduction (*P* = 0.05).

**Conclusions:**

This study presents the first data in the use of a pharmacobehavioral intervention that capitalize on the benefits of open-label placebo and classical drug conditioning for opioid dose reduction in a population with moderate to severe pain exposed to intensive inpatient rehabilitation.

## Abstract 2

Given the complexity of pain management in rehabilitation settings, pharmaco-behavioral interventions can capitalize on the self-modulatory process to enhance the effects of a drug-based intervention.

## 1. Introduction

Patients treated in comprehensive inpatient rehabilitation (CIR) units require optimal pain management to help them achieve their rehabilitation goals.^5^ Inadequately controlled pain during rehabilitation can significantly impact participation in necessary therapy, resulting in decreased patient satisfaction, extended length of stay, and overall morbidity.^7^ Unfortunately, satisfactory pain relief by opioids comes with considerable opioid-related adverse drug events that diminish the quality of life, increase the risk of addiction, and have a detrimental impact on recovery and rehabilitation.

It has been suggested that combining principles of pharmacological conditioning and open-label (honestly prescribed) placebo (OLP) could lower opioid dosage and still provide the same level of pain relief.^4,5^ Conditioning opioid dose extension capitalizes on classical conditioning mechanisms and differential reinforcement rates of the active medication. In a learning phase, the opioid as the unconditioned stimulus is paired with a neutral stimulus—placebo—the neutral stimulus alone becomes a conditioned stimulus, potentially capable of eliciting a conditioned analgesic response after a period of associative learning. Conditioning can be reinforced by adding an olfactory stimulus (smell of a nonfamiliar odor), as the sense of smell is closely aligned with emotional processes, physiologically, and psychologically responses.^8^ Placebo is a complex psychoneurobiological phenomenon, including the release of endogenous substrates that mediate placebo analgesia.^2,7^ This comes from the observation that the opioid antagonist naloxone can efficiently reverse placebo-driven analgesic responses.^5,7^ Several theories try to explain this phenomenon, including treatment expectations, anxiety downregulation, cognitive and emotional processes associated with pain perception, and classical conditioning.^3,7,12^ The use of placebos in the clinical arena represents an ethical challenge because deception or concealment is usually thought to be necessary; OLP bypass ethical issues related to deception or concealment.^2,7^ Open-label placebo involves the presentation of the placebo to the patient and explaining the purpose and possible benefits of this option. Moreover, OLP has already been successfully demonstrated in a pilot randomized controlled trial (RCT) in patients suffering from pain^6,9,10,13^ and nonpain conditions.^1,11,14^

A conditioning OLP (COLP) model can be used to optimize pharmacological treatments without deceiving patients. To date, no study has described or evaluated a COLP intervention in the CIR settings or any RCT concerning pain. Here, we hypothesized that a pharmacobehavioral paradigm would result in decreased opioid utilization by eliciting therapeutic opioid dose extension through placebo-induced analgesia.

## 2. Methods

### 2.1. Subjects

We conducted a pilot RCT in CIR units to explore COLP safety and feasibility. Inpatients from the spinal cord injury and polytrauma units were recruited to participate. Inclusion criteria included (1) hospitalized for intensive rehabilitation and acute pain; (2) spinal cord injury (ASIA A-D); (3) neuropathic and/or nociceptive pain that was moderate to severe (visual analog scale [VAS] score ≥4); (4) on current narcotic use for pain control; and (5) narcotic usage of no more than 120 mg of morphine equivalents or 80 mg of short-acting oxycodone.

### 2.2. Intervention

After informed consent, 20 participants in a 6-day trial were randomized to either COLP or treatment-as-usual (TAU) group. All participants were asked whether they had heard of the “placebo effect” or “conditioning” and then investigators preceded to explain these concepts to both groups. Patients randomized to COLP underwent an acquisition phase of 3 consecutive days where oxycodone (as needed - PRN) intake was paired with a placebo capsule and an odorous stimulus (cardamom oil), followed by 3 days of an evocation phase where oxycodone was completely removed on the fourth and sixth day. During these 2 days, the placebo and odorous stimuli were honestly given in case the patient had pain and requested it. The fifth day of intervention was used as reinforcement of the conditioning, with oxycodone reintroduced to the paradigm. Treatment regime and schedule for the COLP group include the short-acting oxycodone—acquisition and reinforcement days—prescribed on a PRN schedule of 5 to 10 mg, 3 to 4 times per day (without exceeding 80 mg of oxycodone/day). The TAU group received analgesic treatment as prescribed by their treating physicians. Treatment included oxycodone at the standard PRN schedule for both groups. Furthermore, patients in both groups had access to analgesic rescue medication if requested.

### 2.3. Assessments

Patients were evaluated using the following tools: (1) morphine equivalent dose conversion (MEDC) factor is used to standardized opioid usage, having as a reference morphine as the main indicator for analgesic potency, and (2) VAS for pain intensity in the past 24 hours (on day 1 and by the end of the sixth day of intervention). All measurements were performed at baseline and after intervention.

### 2.4. Analysis

Statistical analysis was performed using STATA v.13.1 software (STATA Corp, College Station, TX). Our analysis followed the intention-to-treat principle by last observation carried forward. The primary outcome was total opioid consumption measured by the MEDC, whereas pain control was evaluated with the VAS as a secondary outcome. Within-group comparison from baseline to postintervention was performed using a paired *t* test. Analysis of covariance and linear mixed effects model were used to test the main hypothesis while correcting for baseline differences and covariates.

## 3. Results

A total of 20 patients were enrolled and randomized to participate in this trial. One subject was removed from the study because of increased opioid consumption. All participants tolerated the interventions well; there were no direct side effects associated with the placebo capsule, nor the olfactory stimuli. Table 1 details baseline, demographic, and clinical characteristics.

**Table 1:**
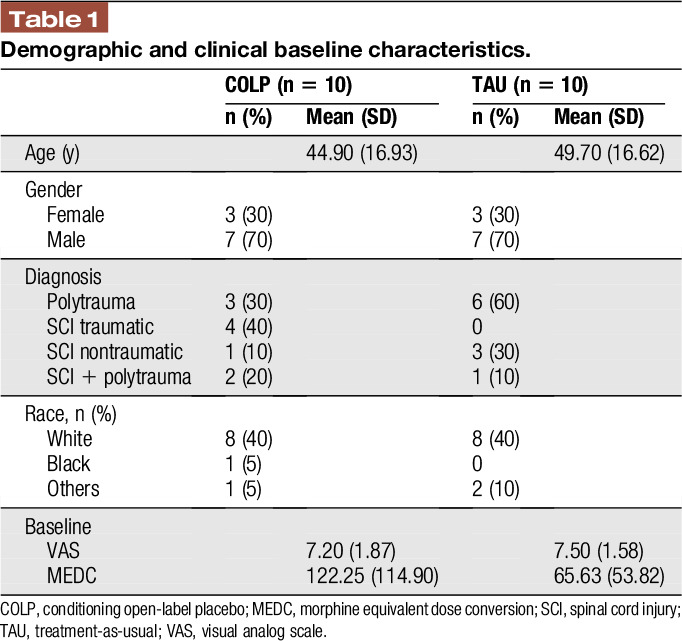
Demographic and clinical baseline characteristics.

### 3.1. Effects of conditioning open-label placebo on total opioid consumption

**Figure 1.**
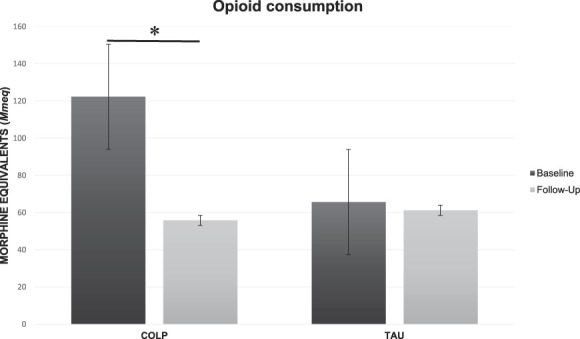
Oxycodone consumption measured in morphine milliequivalents (Mmeq). COLP, conditioning open-label placebo; TAU, treatment-as-usual. *(*P* = 0.0094).

A paired *t* test showed a significant difference in MEDC scores for the COLP group (M = 66, SD = 63, t = 3.29, *P* = 0.0094), opioid dosage decreased from 122.5 mg (SD = 114.9) to 56.25 mg (SD = 60.51), representing a drop of 66% in the total opioid consumption (Fig. 1). After controlling for MEDC differences at baseline, results indicate MEDC scores significantly differed by treatment type after adjusting for preintervention scores, F(2,19) = 29.77 (*P* ≤ 0.001) for the COLP group. No significant difference was observed for the TAU group (*P* ≥ 0.05), opioid use decreased from 65.62 (SD = 53.82) to 61.87 (SD = 58.87), representing a net reduction of 4% by the end of the intervention period.

Linear mixed model analysis (MEDC as a dependent variable with group and time as covariates, and interaction time × treatment) showed time as (z = −4.48, *P* ≤ 0.001) a predictor of MEDC reduction, whereas group (z = −1.66, *P* = 0.097) displayed a marginal effect.

### 3.2. Effects of conditioning open-label placebo on pain

**Figure 2.**
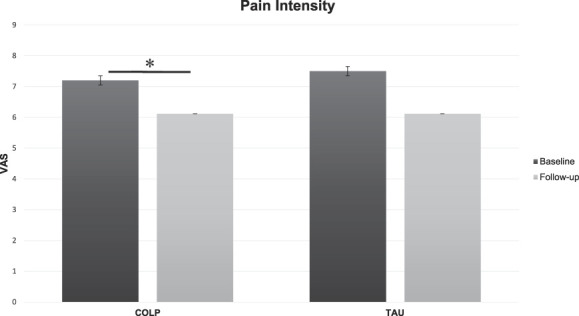
Pain response. COLP, conditioning open-label placebo; TAU, treatment-as-usual; VAS, visual analog scale. *(*P* = 0.005).

In the within-group analysis for VAS scores, results showed a significant effect on pain (Fig. 2) for the COLP group (M = 1.2, SD = 1.03, t = 3.67, *P* = 0.005), whereas a trend was observed for the TAU group (M = 1.2, SD = 1.75, t = 2.16, *P* = 0.05).

## 4. Discussion

Adequate pain management during CIR is imperative. At the forefront of pharmacological treatment for severe pain are analgesic opioids, yet, opioid-based medications now have a negative cloud surrounding them on multiple fronts for both patients and physicians.^16^ In this exploratory trial, our results demonstrated the safety and feasibility of a pharmacobehavioral intervention used in a CIR environment. Results were in line with our proposed hypothesis that participants receiving COLP intervention will have lower opioid consumption while maintaining adequate pain control like those in the TAU group. Given the intervention design, a reduction of 30% was expected in the COLP group; surprisingly, the decrease in MEDC scores went well below this threshold by 66%, and this reduction was significant after adjusting for baseline differences. Pain reduction was similar for both groups (1.2-point difference); however, this must be taken with caution because within-group variability is responsible for this difference.

Many factors are involved in the expression of a placebo through the therapeutic encounter. Conditioning OLP intervention distinctly takes advantage of patients' treatment expectations and likely nonconscious processes involved with classical conditioning. The addition of an olfactory stimulus reinforced the analgesic learned response due to associative learning. Parallel to the pharmacobehavioral strategy, OLP endorsed an honest approach that facilitated patients' understanding of the “self-regulation” concept, as participants were aware of the “inert” nature of placebo while offering an ethical option for the use of COLP as an intervention. Another decisive factor may have been the interaction between patients and clinicians—particularly the nursing team—because they closely monitored the patient's responses to COLP (to insure against poor pain control), highlighting the role of enriched care for patients with severe medical conditions.^16^

There is pressure for pain medicine to shift away from reliance on opioids and ineffective procedures toward comprehensive pain management that includes evidence-based nonpharmacologic options.^15^ Given the complexity of pain management in CIR, there is a need to explore innovative pharmacobehavioral interventions that can take advantage of the self-modulatory process to enhance the effects of a drug-based intervention. Investigations to determine physiological markers of response are further needed. The sample size is the main limitation of this trial; although we did not look for efficacy because of the exploratory nature of the design, our results provide directions for further investigations. Conditioning open-label placebo showed how pharmacotherapy effects can be augmented by associative conditioning enabling opioid “dose extension” through opioid dose reduction. In the future, we intend to perform a longer trial and try to separate the impact of conditioning and OLP involved in this treatment.

## Disclosures

The authors have no conflicts of interest to declare.

This research was supported by funding from the Spaulding Research Catalyst award and the Ellen R. and Melvin J. Gordon Center for the Cure and Treatment of Paralysis. L. Morales-Quezada was funded by the Institutional National Research Service Award from the National Center for Complementary and Integrative Health (NCCIH grant number T32AT000051) and the Foundation for the Science of the Therapeutic Encounter (F-STE).
